# Case Report: Rare case of secondary gastric body tuberculosis—diagnostic challenges arising from the insidious spread of abdominal tuberculosis

**DOI:** 10.3389/fmed.2026.1794381

**Published:** 2026-03-02

**Authors:** Yi-Jing Wang, Jie Cheng, An-Long Wang, Xiao-Wei Qiu, Xiao-Shan Huang

**Affiliations:** 1 Department of Radiology, Hangzhou Red Cross Hospital, Hangzhou, China; 2 Department of Radiology, The Second Affiliated Hospital of Zhejiang Chinese Medical University, Hangzhou, China

**Keywords:** early diagnosis, endoscopy, enhanced CT scan, fine-needle aspiration, gastric tuberculosis

## Abstract

**Background:**

Gastric body tuberculosis is extremely rare and easy to be missed or misdiagnosed.

**Case presentation:**

A 62-year-old female with tuberculous peritonitis was receiving formal antituberculosis treatment, the peritoneal and perihepatic nodules were absorbing during her regular follow-up, but contrast-enhanced CT scan of the abdomen revealed a solid mass on the anterior gastric wall that had penetrated the gastric wall, review her previous enhanced CT scans, we found the gradual extension of an abdominal tuberculous lesion into the gastric wall, which was almost ignored. Endoscopic ultrasound-guided fine-needle aspiration (EUS-FNA) was performed, and finally confirmed by pathology as gastric body tuberculosis.

**Conclusion:**

For patients with perigastric tuberculous lesions, regular follow-up with CT scan is crucial. When necessary, combining gastroscopy with EUS-FNA can help establish an early diagnosis.

## Introduction

Tuberculosis (TB) remains a major global health issue with significant regional heterogeneity. According to the WHO 2025 Global Tuberculosis Report, it is estimated that there will be 10.7 million new cases of TB worldwide in 2024. Of these, approximately 6.5% of the new cases will be in China, ranking 4th among the 30 high-burden TB countries (1). Abdominal tuberculosis, which includes tuberculosis of the gastrointestinal tract, peritoneum, and associated organs, is the sixth most common form of tuberculosis (2). Gastric tuberculosis, whether primary or secondary, is rare, accounting for only 1 to 2% of gastrointestinal tuberculosis cases (3). The majority of gastric tuberculosis cases are secondary, typically resulting from pulmonary tuberculosis or tuberculosis of intra-abdominal organs (4). These cases often occur in the gastric antrum and the lesser curvature near the pylorus, while gastric body tuberculosis is even rarer, with only a few reported cases (5, 6). The clinical presentation of gastric tuberculosis can be asymptomatic or manifest as chronic abdominal pain, bloating, and other symptoms. Some patients may develop vomiting due to concomitant pyloric obstruction. Endoscopic findings often include irregular erosions, ulcers, or submucosal nodules (7, 8), and both clinical and endoscopic manifestations lack specificity. Therefore, even in high-burden tuberculosis areas, gastric tuberculosis may be overlooked and is often not routinely considered by physicians, leading to delays in diagnosis and treatment, or increasing the risk of patients undergoing unnecessary invasive surgeries (9). Early recognition based on ultrasound-guided fine needle aspiration and guideline-concordant therapy are essential to avoid unnecessary surgery and improve outcomes (10). This report aims to present a case of secondary gastric tuberculosis caused by the adjacent invasion of the gastric body wall by an abdominal tuberculosis focus. This case not only illustrates the rarity of gastric tuberculosis but also highlights the insidious progression of a perigastric tuberculosis focus gradually involving the gastric body wall, as seen in sequential follow-up enhanced imaging. By utilizing endoscopic ultrasound-guided fine needle aspiration (EUS-FNA) for diagnosis, this case provides valuable insights for the clinical recognition and diagnosis of similar cases, helping to reduce the risk of missed or misdiagnoses and optimize patient management.

## Case report

A 62-year-old female patient had been undergoing regular follow-up at our Hospital for the treatment of tuberculous peritonitis. Approximately one year ago, the patient began experiencing recurrent abdominal pain and bloating for several months. Gastrointestinal endoscopy suggested a possibility of intestinal tuberculosis. In May 2023, the patient underwent laparoscopy, pelvic adhesion release, and biopsy of the omentum and peritoneal nodules. Postoperatively, the diagnosis of tuberculous peritonitis was confirmed. The patient was then started on a standard anti-tuberculosis treatment regimen (isoniazid, rifampin, ethambutol, and pyrazinamide in combination), along with symptomatic support therapy, including liver protection. Regular outpatient follow-up was scheduled for further monitoring.

During the 13-month postoperative follow-up on June 27, 2024, a contrast-enhanced abdominal CT scan revealed a solid mass on the anterior gastric wall, which had penetrated through the gastric wall, suggesting either tuberculosis involvement or a gastric wall tumor, such as gastric cancer or stromal tumor. The patient presented with the chief complaint of a “gastric mass detected for 2 days.” She did not experience abdominal pain, distension, nausea, vomiting, or other discomforts, and her appetite and general physical condition were normal. Gastroscopy revealed chronic non-atrophic gastritis and a suspicious submucosal mass in the gastric body, prompting further evaluation via endoscopic ultrasound (EUS).

Preoperative blood tests showed a white blood cell count of 2.8 × 10^9/L (Reference range: 3.5–9.5 × 10^9/L) and mildly decreased neutrophil count at 1.59 × 10^9/L (Reference range: 1.8–6.4 × 10^9/L). Except for a mild elevation in CA 72–4 at 10.31 kU/L (Reference range: 0–6.9 kU/L), other tumor markers, including AFP, CA 125, CA 15–3, SCC, CA 19–9, CEA, and CYFRA 21-1, were within normal limits. There were no significant abnormalities in coagulation or biochemical tests.

**Figure 1:**
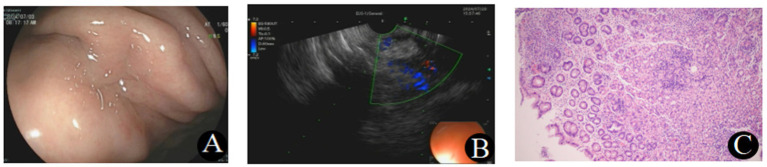
**(A)** Gastroscopy showed chronic non-atrophic gastritis and a suspicious submucosal mass in the gastric body. **(B)** EUS revealed a mass in the greater curvature of the gastric body, with mucosal erosion and uneven internal echo. **(C)** Histopathological examination revealed moderate chronic inflammation with localized epithelioid histiocyte aggregation (H&E, ×40).

EUS showed a mass in the greater curvature of the gastric body, characterized by mucosal erosion, uneven internal echoes, and a lesion size of 2.6 cm × 2.3 cm. Color Doppler imaging revealed no significant blood flow. A COOK 1-22G biopsy needle was used for fine-needle aspiration (FNA), yielding tissue samples from three punctures. Pathology indicated moderate chronic inflammation with localized epithelioid histiocyte aggregation, suggestive of a tuberculous infection. The puncture fluid showed positive acid fast staining, and no malignant cells were detected in the aspirate (Figure 1).

### Imaging examination

A chest CT performed on May 16, 2023, revealed scattered micronodules and fibrotic changes in both lungs. A contrast-enhanced abdominal CT on the same day showed peritoneal thickening with multiple nodules and exudates, consistent with a diagnosis of tuberculous peritonitis.

During the 3-month postoperative follow-up on August 17, 2023, a CT scan showed partial resolution of the peritoneal and mesenteric exudates. However, a new ring-enhancing nodule (1.6 cm × 1.2 cm) was noted adjacent to the anterior gastric wall, though it was not specifically highlighted.

At the 9-month postoperative follow-up on February 27, 2024, CT imaging demonstrated further improvement in the peritoneal and perihepatic nodules. However, the irregular nodule near the anterior gastric wall had grown to 2.6 cm × 2.1 cm and showed close involvement with the gastric wall.

**Figure 2:**
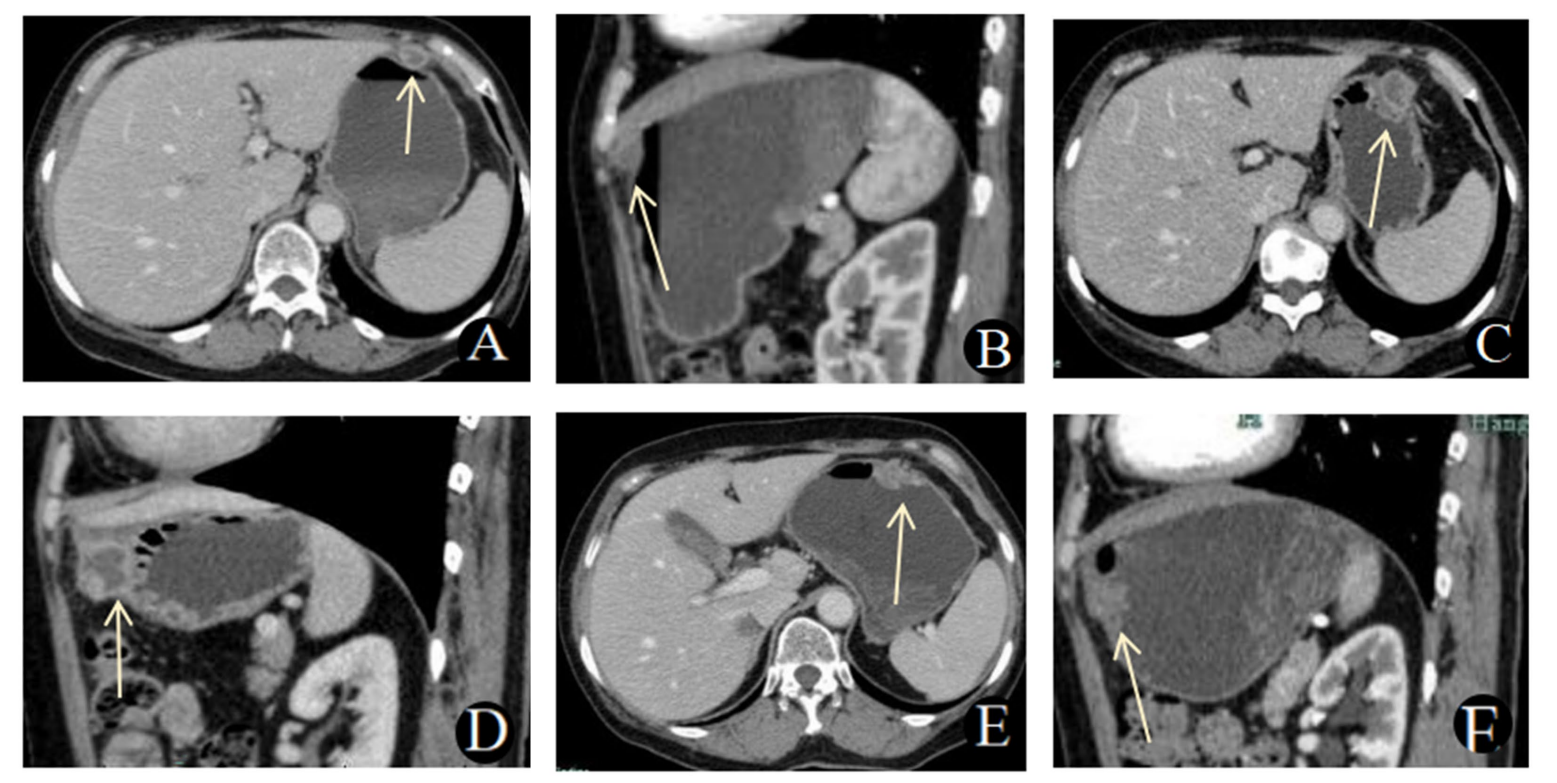
**(A,B)** Contrast-enhanced abdominal CT scan on August 17, 2023. **(A)** Axial CT scan and **(B)** sagittal reconstruction showed a ring-enhancing nodule adjacent to the anterior gastric wall, with the gastric wall remaining intact. **(C-D)** Contrast-enhanced abdominal CT scan on February 27, 2024. **(C)** Axial CT scan and **(D)** sagittal reconstruction revealed that the irregular nodule near the anterior gastric wall had grown larger and involved the gastric wall. **(E,F)** Contrast-enhanced abdominal CT scan on June 27, 2024. **(E)** Axial CT scan and **(F)** sagittal reconstruction showed that the gastric wall lesion had further enlarged and penetrated the gastric wall.

Finally, during the 13-month postoperative follow-up on June 27, 2024, a CT scan revealed further enlargement of the gastric wall lesion to 2.7 cm × 1.7 cm, with penetration through the gastric wall. This raised concern for either tuberculosis involvement or a neoplastic process. The perihepatic nodules remained stable compared to previous scans (Figure 2).

The patient has a history of “diabetes” for over 2 years and is currently taking “Acarbose 1 tablet three times daily and Sitagliptin 1 tablet once daily” with good blood glucose control. She denies any history of hepatitis, pulmonary tuberculosis, or other significant illnesses. Marriage and Reproductive History: She married at the age of 27 and has one daughter and one son, both of whom are in good health, as is her spouse. Family History: Both parents are deceased, but details are unknown. She has five siblings; her eldest sister passed away in a car accident, and the remaining siblings are in good health.

Combined with the patient’s history and pathological findings, asis of gastric tuberculosis was considered. The patient was treated with the HRE antituberculosis regimen, with a total treatment duration of 18 months. At a follow-up visit on September 30, 2024(16 months post-surgery), her blood glucose level was 7.06 mmol/L(Reference range: 3.9–6.1 mmol/L), and liver and kidney function and electrolyte tests were normal. The treatment plan remained unchanged. On January 30, 2026, a follow-up phone call was made. he patient reported that the anti-tuberculosis treatment course had been completed, and she was not currently taking any medication. She returned to her hometown in early 2025, where she has been regularly followed up locally, and her condition remains stable. The patient’s main diagnosis, treatment process, and follow-up timeline are shown in Table 1.

**Table 1:** The patient’s main diagnosis, treatment process, and follow-up timeline.

Relative time	Date	Therapy/Context	Assessment	Key findings	Action/Outcome
Month 0	2023-05-16	At diagnosis of tuberculous peritonitis	Abdominal enhanced CT scan	Peritoneal thickening, multiple nodules/exudation	Laparoscopic biopsy; started HRZE
Month 3	2023-08-17	During anti-TB therapy	Abdominal enhanced CT scan	Peritoneal/mesenteric exudates partially resolved; new ring-enhancing perigastric nodule (1.6 × 1.2 cm), gastric wall intact	Continued therapy; imaging follow-up
Month 9	2024-02-27	During anti-TB therapy	Abdominal enhanced CT scan	Peritoneal/perihepatic lesions improved; perigastric lesion enlarged (2.6 × 2.1 cm), close involvement with gastric wall	Increased suspicion; planned further evaluation
Month 13	2024-06-27	During anti-TB therapy	Abdominal enhanced CT scan	Lesion further enlarged (2.7 × 1.7 cm) with suspected transmural involvement	Gastroscopy + EUS; EUS-FNA performed
Month 13 (same admission)	2024-06-31	Diagnostic work-up	EUS-FNA	Submucosal/perigastric mass; pathology: chronic inflammation + epithelioid histiocytes + caseous necrosis, no malignancy	Diagnosis supported; continued anti-TB
Month 16	2024-09-30	Follow-up	Clinic/labs, NO imagings	Clinical status stable; labs normal (as reported)	Continued regimen/monitoring

## Discussion

Gastrointestinal tuberculosis can occur in various parts of the gastrointestinal tract, with the ileocecal region being the most commonly affected (11). Due to the bactericidal action of gastric acid, the integrity of the gastric wall, and the lack of lymphatic structures in the stomach, gastric tuberculosis is extremely rare (12). The exact mechanism of gastric tuberculosis remains unclear, but three possible routes of infection are widely recognized: (1) direct entry of *Mycobacterium tuberculosis* via contaminated food or sputum into the stomach, infecting the mucosa; (2) hematogenous or lymphatic spread; and (3) direct invasion from adjacent tuberculous lesions (13).

This article reports a rare case of secondary gastric body tuberculosis. Through serial follow-up enhanced CT scans, we present the dynamic radiological evolution of a secondary perigastric tuberculosis focus gradually involving the gastric wall. Initially, the CT scan showed a ring-enhancing lesion near the anterior gastric body wall, with central liquefaction and necrosis, resembling a perihepatic tuberculous abscess. At first, the lesion was not closely related to the gastric wall; however, it gradually exerted pressure on the gastric wall, leading to increased infiltration. Eventually, the lesion extended to the gastric mucosa, with an indistinct boundary between the lesion and the gastric body wall. During follow-up, the patient’s tuberculous peritonitis gradually absorbed and improved, but the lesion on the anterior gastric body wall continued to enlarge. The lesion became more irregular in shape, with a reduction in liquefied necrotic components and an increase in solid components, ultimately forming gastric tuberculosis. Although the more common routes of gastric tuberculosis infection are typically through *Mycobacterium tuberculosis* contaminating food or infected sputum directly entering the gastric mucosa, or via hematogenous/lymphatic dissemination, the continuous imaging changes observed in this case suggest that the mechanism of development is more consistent with secondary gastric tuberculosis caused by direct invasion from an adjacent tuberculous focus.

Previous imaging descriptions of gastric tuberculosis have often focused on the presentation of “gastric wall lesions,” where patients typically show thickening or mass-like changes of the gastric wall at the time of initial discovery (most commonly in the gastric antrum or lesser curvature). Gastric wall thickening is often diffuse and may be accompanied by signs such as gastric distention, fluid retention, and ring-enhancing mesenteric lymph nodes (14–16). In contrast, the initial signs in this case were not significant abnormalities of the gastric wall itself, but rather a ring-enhancing focus adjacent to the anterior gastric body wall with central liquefaction and necrosis. During follow-up, this perigastric lesion gradually enlarged and directly infiltrated the gastric body, eventually forming gastric tuberculosis. The gastric wall showed localized thickening without signs of gastric obstruction, gastric distention, or fluid retention. This represents a significant difference compared to previous reports in the literature.

The clinical manifestations of gastric tuberculosis are non-specific and often lack distinctive features. Some cases may present with gastric outlet obstruction, ulcers, upper gastrointestinal bleeding, or prolonged symptoms of dyspepsia (17). Tuberculous ulcers can quickly form fibrotic scars and firm granulomas, with obstruction more commonly occurring in the gastric antrum or pyloric region. The imaging findings are similarly non-specific, and systematic studies on the CT characteristics of gastric tuberculosis are still relatively limited. Enhanced CT is useful for clearly showing the location of the lesion, its relationship with surrounding structures, and the status of lymph nodes, making it an important tool for the evaluation and follow-up of gastric tuberculosis (13, 18, 19).

Due to the non-specific clinical and imaging findings of gastric tuberculosis, its diagnosis is often challenging and can easily be misdiagnosed as gastric cancer or other diseases (14, 20). Routine gastric biopsy during endoscopy also has significant limitations, as the granulomas in gastric tuberculosis are typically located submucosally, and endoscopic biopsies often sample only the surface mucosa. Deep biopsies can lead to perforation and bleeding, resulting in misdiagnosis or missed diagnosis. In contrast, endoscopic ultrasound-guided fine needle aspiration (EUS-FNA) biopsy plays a crucial role in the diagnosis of gastric tuberculosis. It allows for precise sampling of submucosal or perigastric lesions under real-time imaging guidance, thereby increasing the pathological diagnostic rate and reducing unnecessary surgical interventions to some extent (4, 21). The treatment of gastric tuberculosis primarily involves early and standardized anti-tuberculosis therapy, with a course lasting 12 to 18 months. If complications such as acute massive bleeding (unresponsive to conservative treatment), perforation, obstruction, or difficulty distinguishing from malignant tumors arise, surgical treatment may be considered early.

During the treatment for tuberculous peritonitis, this patient had no significant clinical symptoms. Ultimately, the diagnosis relied on EUS-FNA to obtain tissue samples for pathological examination, along with acid-fast staining of the aspirate. This case highlights that even when abdominal tuberculosis shows an overall trend of improvement, vigilance is needed for the potential progression of localized lesions. Clinical follow-up should systematically assess imaging changes, with particular attention to newly developed or persistently progressing lesions. This approach helps avoid overlooking potential hidden dissemination or local invasion, thereby reducing the risk of missed diagnoses and optimizing treatment decisions.

## Conclusion

Gastric tuberculosis, a rare form of gastrointestinal tuberculosis, presents significant diagnostic challenges due to its non-specific clinical and imaging findings. For patients with a history of tuberculosis who present with gastric outlet obstruction or other unexplained gastric symptoms, gastric tuberculosis should be considered in the differential diagnosis. For patients with abdominal tuberculosis, especially those with or suspected of having perigastric lesions, regular imaging follow-up (such as CT) is crucial. It helps in the timely identification of the subtle progression and invasive trends of the lesions. When necessary, combining gastroscopy with EUS-guided fine needle aspiration (EUS-FNA) can improve the accuracy of sampling and diagnostic efficiency, thereby reducing the risk of misdiagnosis and missed diagnoses and preventing treatment delays.

## Data Availability

The original contributions presented in the study are included in the article/supplementary material, further inquiries can be directed to the corresponding author.
